# Lung nodule localization in hybrid room before minimally invasive thoracic surgery: series of 20 cases and literature review

**DOI:** 10.31744/einstein_journal/2022AO6665

**Published:** 2022-04-07

**Authors:** Guilherme Moratti Gilberto, Priscila Mina Falsarella, Juliano Ribeiro de Andrade, Bruno Pagnin Schmid, Guilherme Cayres Mariotti, Ricardo Mingarini Terra, Jose Ribas Milanez de Campos, Jose Ernesto Succi, Rodrigo Gobbo Garcia

**Affiliations:** 1 Hospital Israelita Albert Einstein São Paulo SP Brazil Hospital Israelita Albert Einstein, São Paulo, SP, Brazil.

**Keywords:** Solitary pulmonary nodule, Lung neoplasms, Thoracic surgery, video-assisted, Preoperative period, Radiology, interventional

## Abstract

**Objective:**

To describe an experience in the preoperative localization of small pulmonary nodules and ground-glass lesions to guide minimally invasive thoracic surgery; in addition, a literature review was conducted, including the main advantages and disadvantages of the different agents used, and site marking in a hybrid operating room.

**Methods:**

A retrospective search was conducted in a Interventional Radiology Department database, between March 2015 and May 2019, to identify patients undergoing preoperative percutaneous marking of lung injuries measuring up to 25mm.

**Results:**

A total of 20 patients were included and submitted to descriptive analysis. All patients were marked in a hybrid room, at the same surgical-anesthetic time. Most often used markers were guidewire, Lipiodol^®^ and microcoils. Despite one case of coil displacement, two cases of pneumothorax, and one case of hypotension after marking, all lesions were identified and resected accordingly from all patients.

**Conclusion:**

Preoperative percutaneous localization of lung injuries in hybrid room is an effective and a safe technique, which can have decisive impact on surgical resection. The choice of marker and of the operating room scenario should be based on availability and experience of service. Multidisciplinary discussions with surgical teams, pathologists, and interventional radiologists are crucial to improve outcome of patients.

## INTRODUCTION

Early-diagnosed small lung lesions, often an incidental finding in screening exams, are usually surgically resected upon presentation.^(1)^ Currently, with the development of video-assisted thoracoscopic surgery (VATS), an important reduction has been observed in morbidity and mortality, recovery time, and length of hospital stay, in addition to better esthetical results, less pain, discomfort, and postoperative complications compared with the conventional open surgery.^(2)^ However, a new challenge had appeared, *i.e.*, the identification of small solid nodules and ground-glass lesions during surgery, particularly smaller than 2cm and apart over 8mm from the pleural surface.^(3)^ These nodules are not visible or palpable on VATS, therefore resulting in conversion rates up to 59% to the conventional open surgery.^(4)^ For this reason, preoperative localization techniques have become of great importance in thoracic surgery, and the interventional radiology has been an essential support for VATS.^(4,5)^

In an attempt to identify these injuries and to guide surgical resection, several localization techniques were described in the literature, with different success rates, complications, related costs, equipment, and skills expected from medical team.^(2)^ The procedure of localization can be done in the preoperative period, divided in different times, or few days before the surgery guided by computed tomography (CT). Localization can be also performed in the perioperative period, prior to surgery (usually in a hybrid operating room) and under the same anesthetic procedure.^(2)^

## OBJECTIVE

To describe an experience in the preoperative localization of small pulmonary nodules and ground-glass lesions to guide minimally invasive thoracic surgery; in addition, a literature review was conducted, including the main advantages and disadvantages of the different agents used, and site marking in a hybrid operating room.

## METHODS

A search was conducted in the Interventional Radiology Department database of a tertiary care medical center in Brazil, to identify patients undergoing preoperative percutaneous localization of lung lesions, measuring up to 25mm, from March 2015 to May 2019. A total of 20 consecutive patients with 24 lesions were included.

### Procedure

The lesions were marked under the same anesthesia employed for surgery in a hybrid operating room, using the device Artis Zeego (Cone-beam CT, Siemens Healthcare GmbH, Erlangen, Germany). Procedures were headed by one experienced Interventional Radiologist, assisted by one Interventional Radiology fellow, followed by surgery performed by the thoracic surgical team with experience in VATS. After anesthetic induction, patients were positioned in lateral decubitus, contralateral to the lesion, a common positioning for VATS. Subsequently, computed tomography imaging acquisition and software programing were conducted under Syngo needle guidance on Syngo X-workstation (Siemens Healthcare). After that, skin entry point and needle path up to the site of interest were programmed. Oriented by laser projections and radioscopy, the needle was placed on the skin entry point and guided up to target lesion.

In the beginning of our experience, lesions were localized with hookwire, commonly used in localization of breast nodules, in association with fatty acid alcohol ester of iodinated poppy seed oil (Lipiodol^®^, Guerbet). Three patients were operated after using this technique; however, after a case of displacement of a fiducial marker, we opted to change the material used. Since then, after discussion with the surgical team, we have adopted 3mm-5.5mm metallic microcoils (radiopaque markers) for all patients and, in some cases, we have combined them with methylene blue.

In cases of very deep lesions, pleural surface localization was conducted with methylene blue to help video identification. For this purpose, after releasing the metallic microcoils, during the return of the 19G needle approximately 5mm to 10mm from the pleural surface, approximately 1mm to 2mm of methylene blue dye were injected in the subpleural lung parenchyma and visceral pleura.

This is a single-center retrospective study approved by the Research Ethics Committee of *Hospital Israelita Albert Einstein* (HIAE), under protocol 4.009.262, CAAE: 29241020.7.0000.0071.

## RESULTS

Patients´ demographics are shown in table 1, and the materials used and their technical aspects are summarized in table 2.

**Table 1:** Demographic characteristics of patients

Patient	Sex	Number of nodules	Size (mm)	Location	CT aspect	Distance to pleural surface (mm)
1	Female	1	17	B6/B10 left	PSN	27
2	Female	1	10	B1 right	PSN	21
3	Male	1	25	B3 right	PSN	15
4	Male	2	A-17 B-7	A: B4 right B: B3 right	PSN	A-29 B-4
5	Male	2	A-8 B-13	A: B4 right B: B3 right	PSN	A-6 B-30
6	Female	3	A-7, B-6, C- 8	A: B1 right B and C: B2 right	Solid	A-21, B-25, C-6
7	Male	1	8	B2 right	Solid	10
8	Male	1	7	B10 right	PGGN	6
9	Male	1	24	B3 left	PGGN	19
10	Female	1	24	B1/B2 left	PGGN	25
11	Female	1	10	B2 rigt	Solid	27
12	Female	1	11	B3 right	Solid	10
13	Female	1	8	B10 left	PGGN	11
14	Female	1	13	B1 right	Solid	31
15	Male	1	9	B2 right	Solid	23
16	Male	1	12	B1/B2 left	Solid	28
17	Female	1	8	B2 right	Solid	17
18	Female	1	8	B1/B2 left	PGGN	24
19	Female	1	10	B1 right	PGGN	21
20	Female	1	21	B3 left	PSN	18

CT: computed tomography; PSN: part solid nodule; PGGN: pure ground-glass nodule.

**Table 2:** Materials and technical aspects

Patient	Materials	Surgery
1	hookwire + Lipiodol^®^, 22G needle	Segmentectomy
2	hookwire + Lipiodol^®^, 22G needle	Wedge resection + 3 margin extending procedures
3	hookwire + Lipiodol^®^, 19G needle	Segmentectomy
4	2 vortex microcoils, 19G needles	A: wedge resection B: lobectomy
5	2 vortex microcoils, 19G needles, Wayne 14F	Segmentectomy
6	2 vortex microcoils, 19G needles	Segmentectomy
7	Vortex microcoil, 19G needles	Segmentectomy
8	Vortex microcoil, 19G needles	Segmentectomy
9	Vortex microcoil, 19G needles	Segmentectomy + lobectomy
10	Hilal microcoil, 19G needles	Segmentectomy
11	Vortex microcoil, 19G needles	Segmentectomy
12	Hilal microcoil, 18G needles	Segmentectomy + lobectomy
13	Hilal microcoil, 19G needles	Segmentectomy
14	Hilal microcoil + Lipiodol^®^ + metilene blue, 20G needle	Segmentectomy + lobectomy
15	Hilal microcoil + metilene blue, 19G needle	Wedge resection
16	Vortex microcoil, 18G needles	Segmentectomy
17	Hilal microcoil, 19G needles	Segmentectomy
18	Hilal microcoil, 19G needles	Segmentectomy
19	2 Hilal microcoils, 19G needles	Segmentectomy
20	Hilal microcoil, 19G needles	Segmentectomy

Lesions were resected accordingly from all patients. Results of pathological anatomy, margins and staging are shown in table 3.

**Table 3:** Pathological anatomy, margins and staging

Patient	Size of lesion* (mm)	Pathology report	Margins	Staging
1	<5	Minimally invasive adenocarcinoma	Free	T1aN0
2	10	Invasive adenocarcinoma	Free	T1aNx
3	21	Minimally invasive adenocarcinoma	Free	T1bNx
4	A: 15 B: 5	A: invasive adenocarcinoma B: minimally invasive adenocarcinoma	Free	T1aN0
5	A: 5,5 B: <5	A: atypical adenomatous hyperplasia B: minimally invasive adenocarcinoma	Free	T1aN0
6	A: 8 B: 6 C: 4	Invasive adenocarcinoma, colorectal metastasis	Free	Mtx
7	10	Organizing pulmonary infarction		
8	7	Lepidic epithelial neoplasia with no interstitial invasion	Free	TisNx
9	16	Differentiated neoplasia, atypical adenomatous hyperplasia	Free	TisN0
10	18	Minimally invasive adenocarcinoma	Free	T1aN0
11	10	Metastasis of mucinous adenocarcinoma of cecal appendix	Free	Mtx
12	13	Epidermoid squamous cell carcinoma	Free	T1aN0
13	11	Adenocarcinoma *in situ*	Free	TisNx
14	4	Invasive adenocarcinoma	Free	T1aN0
15	9	Metastatic colon adenocarcinoma	Free	Mtx
16	7	Invasive adenocarcinoma	Free	T1aNx
17	7	Colloid adenocarcinoma	Free	T1aNx
18	5,5	Adenocarcinoma *in situ*	Free	T1sNx
19	10	Adenocarcinoma *in situ*	Free	T1sNx
20	21	Papillary adenocarcinoma	Compromised margins; new resection: free margins	T1cN0

*Lesion size during anatomopathological analysis.

**Figure 1:**
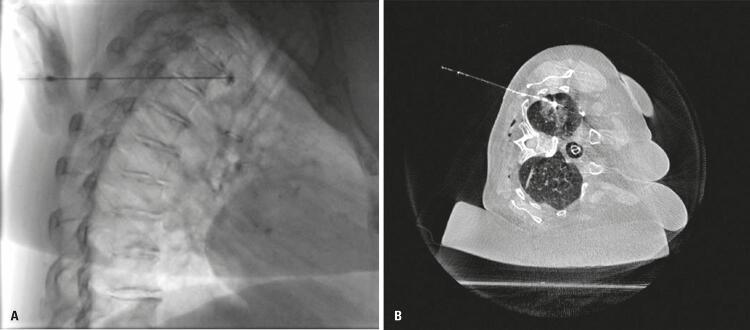
(A) Lipiodol® injection in pulmonary parenchyma guided by cone beam computed tomography and fluoroscopy; (B) Final cone-beam computed tomography showing appropriate Lipiodol® and hookwire location

Patient 2 required extending margins three times for appropriate resection of lesion and to obtain free margins. After the use of hookwire associated with Lipiodol^®^ (Figure 1), the patient presented hypotensive reaction and tremors after the injection of oily drug. Etiologies considered were venous embolization or, less probably, allergic reaction. After this situation, the team opted to discontinue the use of these two markers.

**Figure 2:**
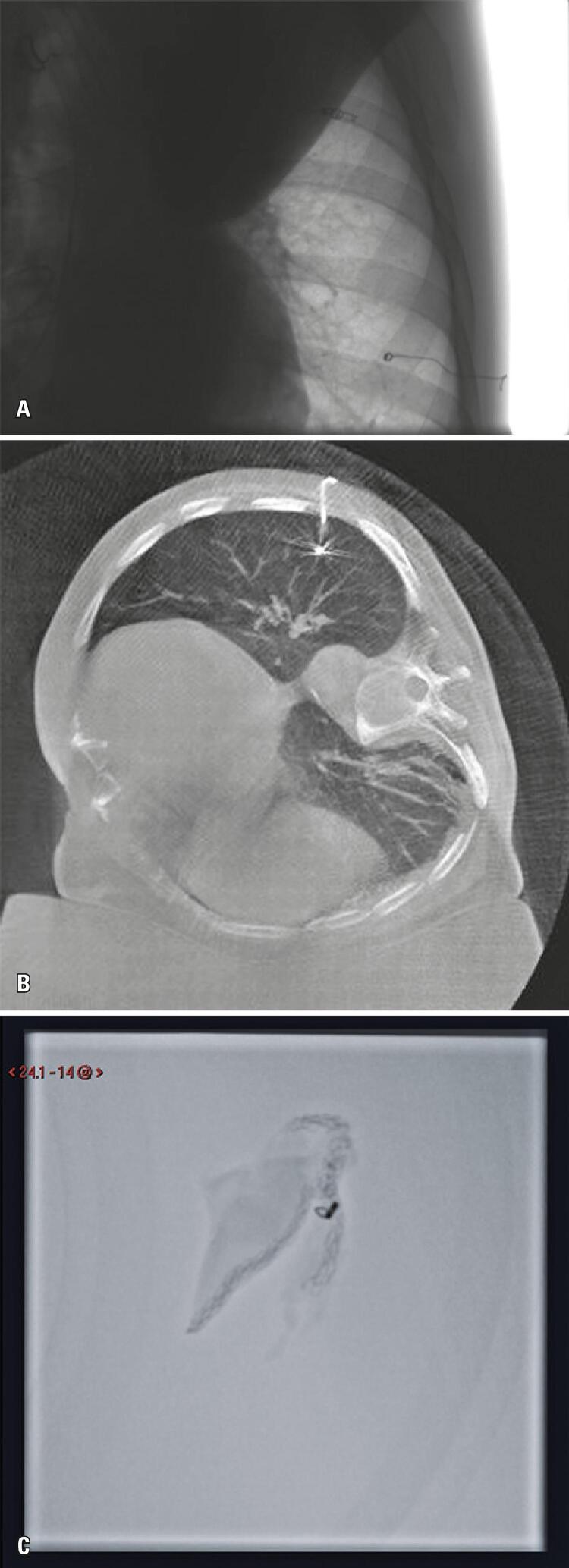
(A) In this case, during the removal of the coaxial needle, part of the coil remained on its interior, which resulted in incomplete formation of the spiral and implant throughout the needle path; (B) In this case, during the removal of the coaxial needle, part of the coil remained in its interior, which resulted in incomplete formation of the spiral and implant throughout the needle path; (C) Microcoil assists pathologist to identify appropriate margins and localization of the lesion in frozen-section biopsy

In the first case in which localization with metallic microcoil was conducted in a hybrid operating room, it was difficult to complete the localization. During the removal of the coaxial needle, part of the coil remained on its interior, which resulted in incomplete formation of the spiral and implant throughout the needle path (Figure 2). However, the surgery had no intercurrent events, and the lesion was completely excised.

**Figure 3:**
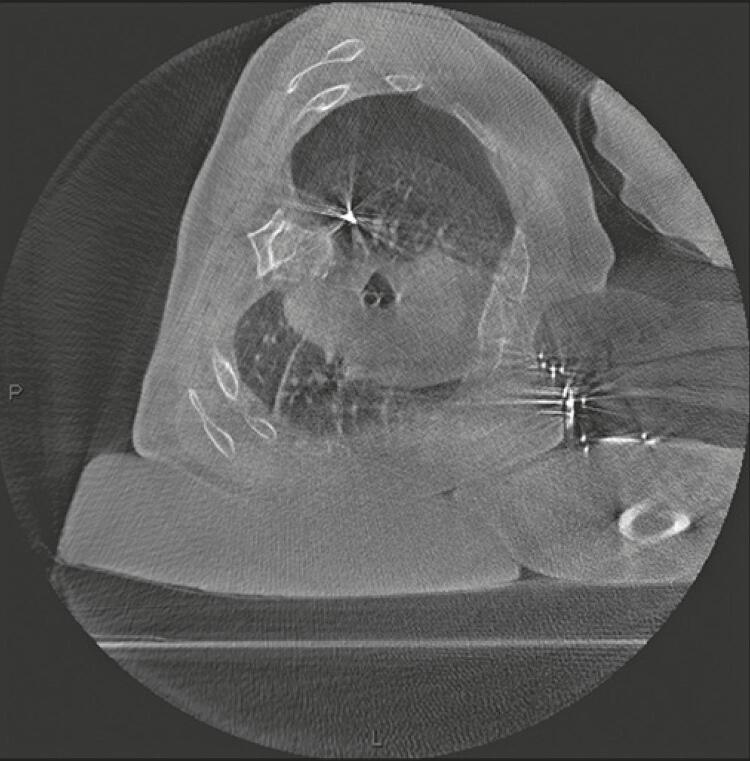
Cone-beam computed tomography demonstrating mild to moderate pneumothorax after microcoil positioning. Pneumothorax had to be drained, once the second lesion localization was impaired by atelectasis

Two patients developed pneumothorax during the localization procedure. One was a patient who would undergo resection of two lung lesions. However, pneumothorax formation conditioned lung atelectasis, hindering visibility and approach to the second lesion; hence, pleural drainage was performed (Figure 3). A pigtail chest tube (14F, Wayne Pneumothorax Set, Cook Medical Inc, Bloomington, IN, United States) was used with complete lung re-expansion, which allowed localization of the second injury and resection. This complication, however, did not affect the surgical result. The second case of complication was in the patient 19; however, at this time, a laminar pneumothorax, which did not require percutaneous drainage. Therefore, surgical resection was conducted with no technical difficulties.

Patient 10 underwent localization with metallic microcoil; nonetheless, due to the difficulties in the perioperative period, it was not possible to appropriately resect the planned segment. This fact was confirmed by radiography of the surgical specimen, which did not identify radiopaque marker; therefore, lobectomy was performed.

Displacement of localization was observed in only one patient (patient 13), who underwent metallic microcoil localization. In this case, the microcoil was implanted on pleural fissure. To perform resection, surgeon used holes on visceral pleural surface to successfully guide the excision. In the last case of the series, patient 20 was also marked with metallic microcoil; however, resection margin was limited and required extending the margin, with no intercurrent events or problems to the patient.

### Literature review

The ideal characteristics of localization marker must include high accuracy; low morbidity and mortality; minimal discomfort to the patient; shorter duration of the procedure; applicability to all lung fields; reproducibility in other environments, specially with less dependence on high-cost specific pieces of equipment; good cost-effectiveness; lower exposition to radiation; and possibility to conduct the procedure in the operating room, not demanding intrahospital transfer.^(2)^

Methylene blue is a cheap, safe, rapid, readily available marker, with high success rates.^(6)^ However, this maker presents the disadvantage of being often dissipated into the normal parenchyma lung and in pleural surface, in addition to be impalpable and difficult to identify in patients with high grade of anthracosis.^(6)^ For this reason, surgery must be conducted within few hours after localization. Some authors have endorsed the use of methylene blue mixed with agar or autologous blood clot, leading to higher concentration of dye in the lesion, and with no leakage.^(1)^ Agar has the advantage of being inert and become palpable, facilitating its localization, in addition to enabling microscopic examination in frozen sections. Nonetheless, it could not be visible in depth injuries at a distance greater than 15mm from the pleural surface, which also require localization of the subpleural lung parenchyma.^(7)^ Rates of detection on pleural surface are up to 97%, and in the nodule up to 93%, therefore, enabling VATS in 90% of patients and risk of pneumothorax of 7%.^(1,7)^ Anaphylaxis secondary to methylene blue is rare, but this complication has been described.^(6)^ Lipiodol^®^ can be used similarly to the methylene blue; however, its use requires intraoperative radioscopy. Success rates are up to 100%, and complications include pneumothorax, hemothorax, air embolism and anaphylaxis.^(8)^

The use of hookwire for preoperative localization is well established for breast procedures. When it comes to small lung injuries, success rates range between 58% and 98%.^(9)^ This is a simple, reproducible, and reliable technique, and probably the most reported and used.^(10)^ Nonetheless, this technique presents higher incidence of complications, being displacement the most relevant one, occurring in up to 47% of cases.^(9,11)^ Displacement can occur at different times, but mainly during transportation to the operating room, lung disinflation in the beginning of VATS, and during surgical manipulation.^(2)^ The higher risk factor for displacement is the short distance from the lesion to the pleural surface, occurring only in 1.9% of lesions apart more than 1cm from the pleura.^(12)^ Other common complications are pneumothorax (7.5% to 40%) and parenchymal hemorrhage (13.9% to 36.0%), which are considered mild complications.^(2)^ The use of double localization hookwire and Lipiodol^®^, or radioisotope, was described with the goal to reduce displacement. Doo et al. observed the use of double localization is safe, has good accuracy and does not increase duration of the procedure.^(13)^ In this series, we begin to use hookwire and Lipiodol^®^, though, due to the high rates of displacement described in the literature, as well as lack of adaptation of involved teams, we opted to change localization technique.

Intraoperative ultrasonography is a non-invasive, rapid, and relatively low-cost procedure to identify non-palpable injuries. On the other hand, it presents considerable technical difficulties in identifying small size nodules. Despite the low rates of complications, the procedure demands operator experience, as well as modern tools and flexible probes, being not very reproducible, and even more limited in cases when achieving total collapse of lungs is not possible, particularly in emphysematous patients.^(4,12,14)^

Radioactive technetium implant to locate small lesions presents high rates of success, as well as relative flexibility for surgical schedule (half-life of 6 hours). This comprises radioisotope emissions of gamma radiation linked to albumin macromolecules, stable on infusion local for up to 24 hours, and localization can be done on the day before the surgery.^(15)^ It requires specific equipment, such as gamma-probe in intraoperative period, which increases costs. This marker presents high success rates, and complications are similar to those associated to several methods.^(2)^ The risk of failure is particularly associated with pleural cavity contamination, mainly in superficial nodules, similarly to dye.^(15)^

Hsu et al. described the use of holes on the visceral pleural with 17G or 20G needle before surgery, as a simple and safe locator for wedge resection by VATS.^(4)^ A pleural puncture was conducted as close as possible to the lesion, without touching the lesion, to avoid path dissemination (seeding). Satisfactory results were obtained in 100% of cases, with no need to convert to thoracotomy; nonetheless, difficulties were observed using the 20G needle.^(4)^ In one of the cases of our series, the thoracic surgery team was guided by holes on visceral pleura with a 19G needle, after displacement of the metallic microcoil to the pleural space (patient 13).

Microcoil localization is precise and effective, and it has the advantages of low rates of displacement and easy identification by radioscopy. The main disadvantage is cost, including the need of fluoroscopy in the operating room, which may not available in some services.^(16)^ Complications rates vary in the literature and the estimates are 9% for pneumothorax, 7% for parenchymal hemorrhage, and 3% to 10% for dislodgment and failure.^(3)^ This marker also helps to evaluate the surgical specimen using radiography, to confirm adequate excision, or for palpation, which help pathologists during frozen section biopsy, in the intraoperative period.^(3)^ When coil is not seen in surgical specimen, it is very likely that surgical margins are compromised, therefore extending margin is recommended, as observed in patient 10. Considering the high rates of success reported associated with advantages of identifying surgical specimen, and good acceptance by surgeons’ team, this method become standard in our service.^(3,8)^

## DISCUSSION

Difficulties in identifying small solid lung nodules or those with ground-glass density during the VATS, led to development of multiple techniques of preoperative localization. Different localization markers have already been described in the literature with a variety of success rates, complications, costs, equipment and experience required. The most often used materials include methylene blue, radiotracer, colored collagen, barium, Lipiodol^®^, intraoperative ultrasonography, hook wires, and metallic microcoils.^(6)^

Few studies have compared the efficacy and risks among different localization techniques. Kleedehn et al., in a retrospective study, compared the use of methylene blue *versus* hookwire, and reported that success rates were similar in both techniques. However, the use of hookwire was associated with higher incidence of complications and displacement in 13% of cases, against no report of leakage of methylene blue.^(17)^ The same study also described a case of air embolism followed by death, after hookwire placement.^(17)^ Nevertheless, we believe risk is inherent to all lung punctures with needles that contain lumen, involving communication from pulmonary venous system with environmental air, regardless of localization marker used.^(18)^

Preoperative localization at CT room can be conducted with local perioperative anesthesia, and this is a minimally invasive procedure. However, it can increase the interval between the procedure and the surgery, therefore resulting in greater chance of complications, such as pneumothorax and displacement.^(2)^

The development and popularization of hybrid rooms allow performing perioperative localization within the same surgical-anesthetic time. In addition, this leads to reduced “risky period” to patients, which is characterized by the interval between two procedures with consequent lower morbidity.^(19,20)^ There are lower rates of displacement and pneumothorax, but no differences in hemorrhagic complications, nor in length of hospital stay, or success rates.^(2,19,20)^ In addition, in a hybrid room, if localization failure is suspected, a new tomography can be carried out to provide information and also enable a new implant procedure.^(2)^

Preoperative percutaneous localization of lung lesions in hybrid room is an effective and a safe method, which can have decisive impact on surgical resection, with low rates of complications.

The limitations of the present study include its retrospective analysis of data and selective single-center experience, with a small number of patients. On the other hand, the different marking techniques presented showed encouraging results, since all lesions were identified and resected accordingly. In our service, due to the experience we have acquired along with the thoracic surgical team, procedures are conducted in hybrid room, and coils are preferred. By adopting these guidelines, we observed less complications, such as displacements, and high successful surgery rates.

## CONCLUSION

Microcoil localization in hybrid room showed promising results. However, the selection of markers may be based on availability and experience of service, and is usually made after discussions with multidisciplinary teams including surgeons, pathologists, and interventional radiologists.
